# The mediating role of social media addiction and phubbing in basic psychological needs in relationships and relationship satisfaction

**DOI:** 10.3389/fpsyg.2024.1291638

**Published:** 2024-03-22

**Authors:** Hüseyin Buğra Karaman, Coşkun Arslan

**Affiliations:** Department of Psychological Counseling and Guidance, Necmettin Erbakan University, Konya, Türkiye

**Keywords:** romantic relationships, basic psychological needs, relationship satisfaction, phubbing, social media addiction

## Abstract

This study aimed to examine the mediating role of phubbing and social media addiction in the correlation between satisfaction levels of basic psychological needs in romantic relationships and relationship satisfaction. The participants were 958 students from various universities in Türkiye. The research utilized demographic information form for personal information of the participants, the Satisfaction of Basic Psychological Needs in Romantic Relationships Scale, the Generic Scale of Phubbing, the Social Media Addiction Scale-Adult Form, and the Relationship Satisfaction Scale. It was concluded in the research that social media addiction and phubbing had a mediating role in the correlation between the needs for love/belonging *β* = 0.05, power *β* = 0.03, and freedom *β* = −0.08 and the relationship satisfaction. The bootstrapping method performed in the study showed that indirect effect of the need for fun on the relationship satisfaction was significant, but in the Sobel test, social media addiction and phubbing was not found to have a mediating role in this correlation (*p* > 0.05). It was concluded that phubbing had a mediating role for all needs other than the need for fun in the correlation between satisfaction of basic psychological needs in romantic relationships and social media addiction. Finally, social media addiction was found to have a mediating role in the correlation between phubbing and relationship satisfaction. These findings were discussed in line with the literature. As shown by the findings, it was observed that satisfaction of basic psychological needs in romantic relationships affected the relationship satisfaction, and social media addiction and phubbing had a mediating role in that correlation.

## Introduction

In Erikson’s stages of psychosocial development, young adulthood is called “intimacy versus isolation.” During this stage, young adults want to be in a special relationship in which they can develop intimacy and emotionally mature. When they fail to do so, they come up against emotional isolation. Although there are some benefits to remain single during this stage, in the long run, individuals might be at risk in the matters of emotional maturation and happiness (Burger, 2006). It was emphasized that Erikson based these stages on the epigenetic principle, and that individuals’ success in these stages will be important for their healthy progress in other ones (Çapri, 2020). Relationship satisfaction is an important subject in the assessment of behaviors, emotions, and thoughts in a relationship (Hendrick, 1988). It involves subjective assessments of individuals regarding their relationships and offers a viewpoint of their partner and the relationship with them (Hendrick et al., 1998). In another definition, relationship satisfaction is described as the extent of being pleased and satisfied with one’s own relationship and as a strong indicator of a long and successful relationship (Anderson and Emmers-Sommer, 2006). There can be several factors that impact relationship satisfaction. Considering other variables that affect relationship satisfaction, a negative correlation was suggested between relationship satisfaction and economic dependence for women and lack of sex life for men. Relationship satisfaction for both genders was found to positively correlated with factors such as length of relationship, emotional commitment, and relationship awareness but negatively correlated with negative conflicts (Frazier and Esterly, 1990). It has been stated that the concept of relationship satisfaction plays a key role in understanding how marriages work, in studies on relationships, and in the literature on the improvement of marriages (Funk and Rogge, 2007; Graham et al., 2011).

Relationship satisfaction can be affected by several situations. One of these situations is whether individuals in a relationship are healthy. The definition of a healthy individual is addressed differently in theories. Freud, the founder of the psychoanalytic theory, defines the healthy individual as an individual who is able to love and work. Adler describes the healthy individual as an individual who can benefit the society which they live in. Based on Maslow’s (1998) hierarchy of needs, the person-centered approach addresses the healthy individual as a person who live a life that satisfies their needs in balance (Murdock, 2013). When examining the changes in approaches to healthy individual historically, it was emphasized by Glasser that individuals’ mental health might be negatively affected by failure to satisfy their basic needs (Glasser, 1965, 1998; Wubbolding, 2015). In previous studies, basic psychological needs have been found to be correlated with well-being (Cihangir-Çankaya, 2009), physical, verbal violence (Movehedirad et al., 2021), subjective well-being (Türkdoğan and Duru, 2012), bullying (Eşici, 2013), social media addiction (Bozkurt and Bozkurt, 2022), internet addiction (Eyyüpoğlu and Özbay, 2018; Sever, 2021), anxiety (Erden, 2021), and marriage satisfaction (Moblian et al., 2021).

There are five basic needs with four of them being psychological, and one of them being physiological (Glasser, 1998), which are survival, love/belonging, power, freedom, and fun. Survival; humans, like all living beings, strive to survive. Their struggle may include hard work, not quitting, providing their own security, and reproduction. In addition, what differentiates between humans and animals is that individuals live in the manner that they carry themselves into future. Love/belonging; Glasser put the first emphasis on the need for loving and being loved. As stated by him, this need plays a key role in our family relations, friendships, and romantic relationships throughout our life. He explained that satisfying this need affects how valuable individuals feel. Need for power; power is defined as a human-specific need. How one is not content with what they own and want more is described as a behavior specific to humans. It has been stated that animals are not aggressive when they have enough to eat whereas humans are not content with what they already have and ask for more despite knowing that it will cause others to have less. Need for freedom; the leading situation which harms the need for freedom is psychology of external control. It has been emphasized that the first need that individuals are concerned about when they feel externally controlled is the need for freedom Individuals may have difficulty expressing themselves, making decisions and choices when they lose that need. Need for fun; it has been addressed as a product of learning. Humans are beings that have fun throughout their lives. From the behavioral point of view, laughing has been described as the behavior that reflects having fun best. Humans have found a way to entertain themselves from birth till death. It is the easiest need to satisfy for individuals (Glasser, 1965, 1998; Gray-Little and Burks, 1983; Wubbolding, 2015).

In the case of the basic needs and relationships together, Glasser stated that the need for power damages the relationships. (Glasser (1998)) emphasized the importance that couples steer their needs together. When satisfying their needs, individuals need a person or group to whom they feel emotionally close within their lives. However, living their lives without being aware of their needs may cause individuals to have difficulties satisfying those needs. Glasser therefore highlighted that individuals should be informed of the needs from an early age; otherwise, they might suffer throughout their lives, and such suffering might cause them to find unrealistic ways to meet their needs (Glasser, 1965, 1976, 1998; Wubbolding, 2015).

Addressing the need for loving and being as one of the most important needs as well as attaching importance to balanced satisfaction of needs show us the importance of close relationships (Glasser, 1965). According to Glasser (1998), individuals can only notice which need has not been satisfied when a problem occurs once they have knowledge about those needs. It is likely that the individual becomes happy when those needs are met. However, if those needs are not satisfied, the individual could choose either effective mental health behaviors or ineffective mental behaviors. Ineffective mental health behaviors involve negative addictions (drugs, alcohol, eating, gambling, etc.) and adverse symptoms (Wubbolding, 2015; Bozkurt and Bozkurt, 2022). Despite not being mentioned under negative addictions in the sources, social media addiction and phubbing, which are increasingly given place in research studies, can be handled as two of the other ineffective mental health behaviors in individuals.

Phubbing is described as directing one’s attention to their smartphone during the act of communicating with one person or more. The term “phubber” is defined as the person who chooses their smartphone over communicating with the other person, and the term “phubbee” is defined as the person who finds themselves alone while others are engaged with their smartphones (Chotpitayasunondh and Douglas, 2016). Phubbing is also described as preferring to pay attention to one’s own smartphone while communicating with someone, and therefore, halting the communication. Phubbing has become inevitable because individuals have their smartphones with them in every area of life (Roberts and David, 2016).

With increased frequency of using smartphones and the Internet, many applications are now implemented in the smartphones. Individuals can occupy themselves with their smartphones in many activities within their lives. Such occupation may include messaging, browsing, and social media (Nazir and Pişkin, 2016; Cizmeci, 2017). All these indicate that smartphones harbor several elements which trigger phubbing. Karadağ et al. (2016) considered smartphones, social media, Internet, digital games, and application addiction as phubbing-related concepts. Phubbing alone can be handled as an addiction whereas social media, Internet, digital games, and applications may increase the smartphone use. In another study, phubbing was addressed as SMS addiction, smartphone addiction, social media addiction, Internet addiction, and video game addiction (Al-Saggaf et al., 2019).

Social media addiction can be described as using social media networks to the extent that they affect one’s daily life. It is known that social media networks are extensively used especially among adolescents and young people. In the Statista (2020) report, it was concluded that 3.6 billion individuals used social media networks globally. In the same report, it was stated that the most used social media network was Facebook with 2.7 billion users. Social media networks such as YouTube and WhatsApp were also observed to be among the most used networks. The report issued by the platform called We Are Social (2020) stated that there were 54 million social media network users in Türkiye. It was listed in the same report that the most used social media networks were YouTube, Instagram, and WhatsApp, respectively. As can be found in the 2022 report of the said study, overall population increased by about two million; however, there were approximately 69 million social media network users (We Are Social, 2022). In a study, Instagram usage was ranked first among social media networks with 69.9%, which was followed by YouTube and Facebook, respectively (Özdemir, 2019).

Studies showed a negative significant correlation between social media addiction and relationship satisfaction (Porter et al., 2012). Similarly, there are research studies that found negative significant correlations between phubbing and relationship satisfaction (Roberts and David, 2016; Cizmeci, 2017; Wang et al., 2017; Chotpitayasunondh and Douglas, 2018a; Lapierre and Custer, 2020). In another study, it was found that men had higher scores of problematic Internet use than the women, and there was a negative significant correlation between the scores of problematic Internet use, its subscales excessive use and social benefit and marriage satisfaction (Aylıkçı, 2018). Other studies could not find significant correlations between social media addiction and relationship satisfaction (Goodman-Deane et al., 2016; Kılıç, 2020). However, negative correlations between social media addiction and phubbing and relationship satisfaction were observed in some of the studies.

Based on the totality of information above, the present study examined the mediating roles of social media addiction and phubbing in the correlation between individuals’ satisfaction levels of basic psychological needs in romantic relationships and their relationship satisfaction levels. To that end, a structural equation model was generated and tested. Glasser emphasized that individuals may develop ineffective mental health behaviors and addictions when their basic psychological needs are not satisfied. In addition, it was emphasized that the relationships in the lives of individuals play an important role in the satisfaction of basic psychological needs. Therefore, in this study, the mediating role of social media addiction and phubbing in the relationship between the satisfaction of individuals’ basic psychological needs and relationship satisfaction was examined. In line with the purpose of the research, answers were sought to the following questions:

(1) Is there a relationship between basic psychological needs, phubbing, social media addiction and relationship satisfaction?
(2) Does social media addiction have a mediating role in the relationship between basic psychological needs and relationship satisfaction?
(3) Does phubbing have a mediating role in the relationship between basic psychological needs and relationship satisfaction?
(4) Do social media addiction and phubbing have a mediating role in the relationship between basic psychological needs and relationship satisfaction?

## Method

### Research model

The research was conducted in the correlational survey model. The aim with this type of model is to test the level of change between the variables. Correlational research studies also aim to reach larger sample groups and generalize the research data to the population more conveniently.

### Participants

For the research, individuals in young adulthood were studied. University students who are in a current relationship or have had a relationship before were included in the research. After the ethics committee permissions, research permissions were obtained from the universities where the research was planned to be conducted and data were collected. Nine hundred and fifty-eight undergraduates including 765 women (79.9%) and 193 men (20.1%) were enrolled in the research as participants. Ages of the participants ranged between 17 and 29 years, and their mean age was 21.10 years (*M* = 21.10, *SD* = 1.92).

### Data collection process

A request for ethical committee approval was filed to Necmettin Erbakan University Social Sciences and Humanities Ethical Committee before collecting data from the patients face-to-face. The committee granted approval for the research in the meeting dated 15.10.2021 and numbered 09 with the decision no. 2021/511. Following the approval, research permits were obtained from the relevant bodies. Next, research data were collected from young adults in the fall semester of 2022–2023. Before starting the research, participants were informed about the declaration of volunteering. Participants who stated that they were not volunteers were not included in the study. Since the research was conducted with individuals in young adulthood, university students were included in the research.

### Data collection instruments

#### Satisfaction of basic psychological needs in romantic relationships scale

The scale was developed by Karaman and Arslan (2023). Satisfaction of Basic Psychological Needs in Romantic Relationships Scale was designed to evaluate basic psychological needs in Glasser’s Choice Theory (1965) through relationships. In the Exploratory Factor Analysis performed, a four-factor structure explaining 54% of total variance was obtained. Examples of items in the scale are “I make decisions about our relationship jointly with my partner” and “I feel that my partner loves me in my relationship.” The Confirmatory Factor Analysis was performed on two levels: Based on the first-level CFA results, the fit indices were found to be χ2/sd = 2.32, GFI = 0.91, CFI = 0.96, AGFI = 0.87, RMSEA = 0.07, SRMR = 0.05, and NFI = 0.93. According to the second-level CFA results, the fit indices were observed to be χ2/sd = 2.33, GFI = 0.90, CFI = 0.95, AGFI = 0.87, RMSEA = 0.07, SRMR = 0.05, and NFI = 0.92. In the concurrent validity studies performed, Basic Psychological Need Satisfaction Scale – Relationship Domain was utilized, and a positive significant correlation was found between the two scales (*r* = 0.75, *p* < 0.01). The Cronbach’s alphas for the total scale and its subscales were calculated to be 0.94 for the total scale, 0.93 for the need for love/belonging, 0.89 the need for power, 0.82 for the need for freedom, and 0.86 for the need for fun. The item-total correlation values were found to range between 0.49 and 0.84. For the test–retest studies, the Satisfaction of Basic Psychological Needs in Romantic Relationships Scale was applied to a total of 71 patients 3 weeks apart. The participants were asked to write down nicknames in their application forms. The test–retest reliability coefficient was calculated to be 0.81. In the case of the present research, the Cronbach’s alphas for the subscales were calculated to be 0.90 for the need for love/belonging, 0.86 the need for power, 0.76 for the need for freedom, and 0.81 for the need for fun. A Cronbach’s alpha of 0.91 was calculated for the total scale.

#### Generic scale of phubbing and generic scale of being phubbed

The scales were developed by Chotpitayasunondh and Douglas (2018b). The adaptation studies for the Turkish language were performed by Orhan-Göksün (2019). The Generic Scale of Phubbing (GSP) and the Generic Scale of Being Phubbed (GSBP) were adapted to the Turkish language in one scale. GSP is a 15-item scale whereas GSBP consists of 22 items. Examples of items in the GSP are “I feel anxious if my phone is not nearby” and “I get irritated if others ask me to get off my phone and talk to them.” It is a Likert scale rated from “never” to “always.” The confirmatory factor analysis in the adaptation studies was conducted with a total of 180 participants. Four-factor structure of GSP (χ2/sd = 1.99, *p* ≤ 0.001, RMSEA = 0.07, SRMR = 0.06, NFI = 0.92, CFI = 0.96, GFI = 0.89) and three-factor structure of GSBP (χ2/sd = 2.04, *p* ≤ 0.001, RMSEA = 0.08, SRMR = 0.07, NFI = 0.90, CFI = 0.95, GFI = 0.82) was confirmed. In the present study, GSP scale of the two scales was utilized. Cronbach’s alphas were calculated for the total scale and subscales of GSP in the research. Cronbach’s alphas of 0.82, 0.78, 0.90, and 0.77 were found for the subscales nomophobia, interpersonal conflict, self-isolation, and problem acknowledgement, respectively. Cronbach’s alpha of the total scale was calculated to be 0.88.

#### Social media addiction scale – adult form

Validity and reliability studies were conducted with 1,047 adults for the Social Media Addiction Scale-Adult Form (SMAS-AF) developed by Şahin and Yağcı (2017). The 5-point Likert scale (not applicable at all – strongly applicable) comprises 20 items and two subscales (virtual tolerance and virtual communication). Examples of items in the scale are “The first thing I do when I wake up in the morning is go on social media” and “I neglect my family members because of social media.” Factor loadings of the subscales were found to range between 0.61 and 0.87. Cronbach’s alphas for the total scale and the subscales virtual tolerance and virtual communication were found to be 0.94, 0.92, and 0.91, respectively. In a study to confirm the two factors, the fit indices were calculated to be χ^2^/sd = 3.05, RMSA = 0.06, SRMR = 0.06, NFI = 0.59, CFI = 0.96, GFI = 0.90, and AGFI = 0.88 and found to be acceptable. Test–retest reliability coefficients were calculated to be 0.93 for the total scale, 0.91 for virtual tolerance, and 0.90 for virtual communication. Cronbach’s alphas of 0.81 and 0.79 were found for the SMAS-AF subscales virtual tolerance and virtual communication, respectively. For the total scale, a Cronbach’s alpha of 0.87 was calculated.

#### Relationship satisfaction scale

Developed by Hendrick (1988), the scale was adapted into Turkish language by Curun (2001). It is a seven-item, one-factor, seven-point Likert scale. Fourth and seventh items of the scale are reverse-coded. Examples of items in the scale are “How well does your partner meet your needs?” and “How many problems are there in relationship?.” The scale was found to be valid and reliable for measuring the relationship satisfaction in the Turkish adaptation study. The scale was found to have a one-factor structure in that study, and its Cronbach’s alpha was calculated to be 0.86. The Cronbach’s alpha calculated for the one-factor structure of the scale is 0.90 for the present research.

### Data analysis

The structural equation model is a statistical method that has become even more important for social sciences in recent years. AMOS 24 was used in the analyses. What makes this statistical method important is that it can analyze multiple variables and latent factor structures simultaneously. Thus, ability to test a model as a whole rather than calculating the paths between variables one by one can provide researchers with more information on the model. Also, the feature of confirmation rather than explanation can provide more precise results in the testing of hypotheses. Causal relationship and ability to examine the roles of mediator variables are among important features of the method. The structural equation model has been described as a powerful method as it is able to study the relationships among several variables (Kline, 2015; Byrne, 2016).

Before the model analysis, required conditions for the structural equation model were investigated. A missing data analysis was performed, and values were imputed for the missing data the with mean imputation method. An outlier analysis was performed based on *Z* scores, and the outliers were excluded from the dataset. Normal distribution was tested for latent and indicator variables, and values were found to range between −2 and +2 skewness-kurtosis values (George and Mallery, 2010). Analyses were conducted for the relationship anticipated between the latent and indicator variables in the model with the Pearson’s Product–Moment Correlation Coefficient method. The maximum likelihood method was used in the analyses since the sample was of sufficient size and the data were univariate normally distributed. For the model fit, chi-square (χ^2^), RMSEA, CFI, GFI, SRMR, and AGFI values were examined. These fit indices have been suggested to be the most reported indices (Sümer, 2000; Tabachnick and Fidell, 2015).

## Findings

### Preliminary analyses, testing of normality, and inter-variable correlations

Before testing the model established in the research, preliminary analyses were completed for the purposes of structural equation model.

Normal distribution of the latent and indicator variables was tested, and their skewness and kurtosis values were found to range between −2 and +2 (George and Mallery, 2010). Information on those values is provided in Table 1. In addition to this, when the univariate normality results were examined through the AMOS program, it was seen that each of them was between −2 and +2 skewness kurtosis values.

**Table 1:** Skewness-kurtosis values of the latent and indicator variables.

Variable	Skewness	Kurtosis
Love/belonging	−1.03	0.29
Power	−1.11	0.84
Freedom	−1.42	1.99
Fun	−0.72	−0.19
Social media addiction	0.09	−0.10
Phubbing	0.57	0.04
Satisfaction relationship	−0.61	−0.45

As shown in Table 2, significant positive correlations were found among the need for belonging of the basic psychological needs and other subscales of basic psychological needs (*r* = 0.68, *r* = 0.37, *r* = 0.56; *p* < 0.01). Significant negative correlations were found between the need for love/belonging and social media addiction and phubbing (*r* = −0.15, *r* = −0.10; *p* < 0.01). A significant positive correlation was found between the need for love/belonging and relationship satisfaction (*r* = 0.73; *p* < 0.01).

**Table 2:** Inter-variable correlations.

Variable	1	2	3	4	5	6	7
Love/belonging	1						
Power	0.68^**^	1					
Freedom	0.37^**^	0.53^**^	1				
Fun	0.56^**^	0.59^**^	0.36^**^	1			
Social media addiction	−0.15^**^	−0.13^**^	−0.07^*^	−0.04	1		
Phubbing	−0.10^**^	−0.10^**^	−0.13^**^	−0.06^*^	0.70^**^	1	
Satisfaction relationship	0.73^**^	0.67^**^	0.37^**^	0.57^**^	−0.16^**^	−0.11^**^	1

^*^p < 0.05, ^**^p < 0.01.

Significant positive correlations were observed between the need for power and the needs for freedom and fun of the basic psychological needs (*r* = 0.53, *r* = 0.59; *p* < 0.01). The need for power was found to be significantly negatively correlated with social media addiction and phubbing (*r* = −0.13, *r* = −0.10; *p* < 0.01) and significantly positively correlated with relationship satisfaction (*r* = 0.67).

The needs for freedom and fun of the basic psychological needs were found to be significantly positively correlated (*r* = 0.36; *p* < 0.01). The need for freedom was found to be significantly negatively correlated with social media addiction and phubbing (*r* = −0.07; *p* < 0.05; *r* = −0.13; *p* < 0.01) and significantly positively correlated with relationship satisfaction (*r* = 0.37, *p* < 0.01).

The need for fun was found to be significantly negatively correlated with phubbing (*r* = −0.06; *p* < 0.05) and significantly positively correlated with relationship satisfaction (*r* = 0.57). No significant correlation could be found between the need for fun and social media addiction (*p* > 0.05).

Social media addiction was found to be significantly positively correlated with phubbing (*r* = 0.70; *p* < 0.01) and significantly negatively correlated with relationship satisfaction (*r* = −0.16; *p* < 0.01). Finally, a significant negative correlation was found between phubbing and relationship satisfaction (*r* = −0.11; *p* < 0.01).

Chi-square (χ^2^) was calculated to be 5.027 for the fit indices. Values below 5 can be considered acceptable. It is a fit value that is affected by sample size (Gürbüz, 2019).

**Figure 1:**
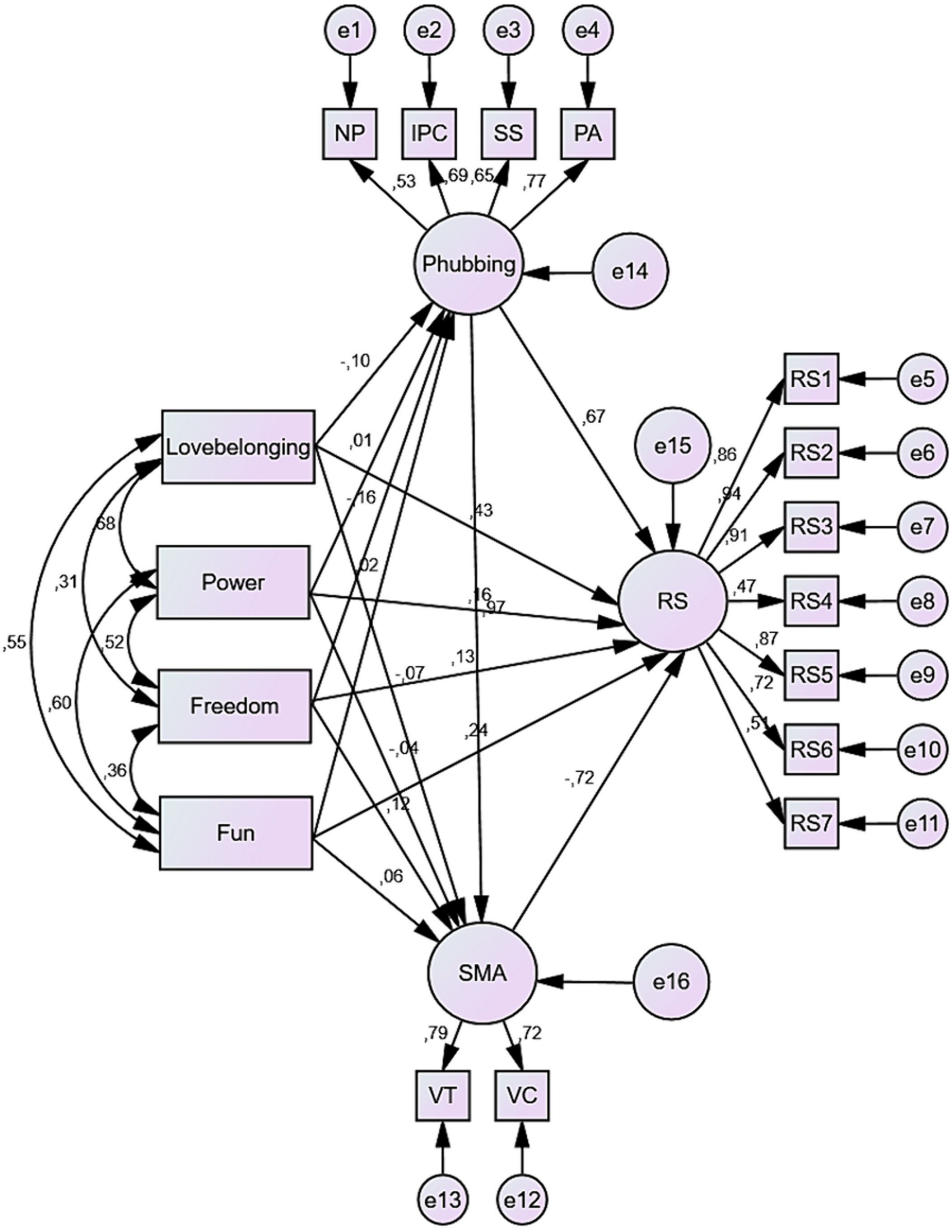
Standardized path coefficients for the structural model (NP, nomophobia; IPC, interpersonal conflict; SS, self solation; PA, problem acknowledgement; RS, relationship satisfaction; SMA, social media addiction; VT, virtual tolerance; VC, virtual communication).

As for the other fit indices in the table, the model was found to have the following fit indices: standardized RMR (SRMR) = 0.0431; perfect fit, GFI = 0.94; good fit, AGFI = 0.91; good fit, RMSEA = 0.07; good fit, CFI = 0.96; perfect fit, and NFI = 0.95; perfect fit (Browne and Cudeck, 1993; Hu and Bentler, 1999; Sümer, 2000; Thompson, 2000; Loehlin, 2004; Kline, 2015; Tabachnick and Fidell, 2015; Çokluk et al., 2018) (Figure 1) and (Table 3).

**Table 3:** Model fit indices.

Fit indices	Good fit values	Perfect fit values	Model fit indices
Chi-square (χ^2^)	2 < χ^2^ < 3	0 < χ^2^ < 2	5.027
GFI	0.90 < GFI < 0.95	0.95 < GFI < 1.00	0.94
AGFI	0.90 < AGFI<0.95	0.95 < AGFI<1.00	0.91
CFI	0.90 < CFI < 0.95	0.95 < CFI < 1.00	0.96
NFI	0.90 < NFI < 0.95	0.95 < NFI < 1.00	0.95
RMSEA	0.05 < RMSEA<0.08	0.00 < RMSEA<0.05	0.07
SRMR	0.05 < SRMR<0.08	0.00 < SRMR<0.05	0.04

### Direct effects in the model

Considering the direct effects in the model, an increase of one unit in the need for love/belonging of the basic psychological led to an increase of 0.14 in relationship satisfaction (*β* = 0.43). As for the other needs, the need for power, the need for freedom, and the need for fun led to increases of 0.05 (*β* = 0.16), 0.05 (*β* = 0.13), and 0.08 (*β* = 0.24), respectively, in relationship satisfaction (Table 4).

**Table 4:** Path coefficients for the model.

Variables	Non-standardized coefficients	Standardized coefficients	*p*
Love/belonging → Phubbing	−0.07	−0.10	0.050
Power → Phubbing	0.01	0.01	0.803
Freedom → Phubbing	−0.15	−0.16	0.000
Fun → Phubbing	0.02	0.02	0.611
Phubbing → Social media addiction	1.27	0.97	0.000
Love/belonging → Social media addiction	−0.06	−0.06	0.085
Power → Social media addiction	−0.04	−0.04	0.378
Freedom → Social media addiction	0.15	0.12	0.000
Fun → Social media addiction	0.06	0.06	0.091
Love/belonging → Relationship satisfaction	0.14	0.43	0.000
Power → Relationship satisfaction	0.05	0.16	0.000
Freedom → Relationship satisfaction	0.05	0.13	0.041
Fun → Relationship satisfaction	0.08	0.24	0.000
Social media addiction → Relationship satisfaction	−0.23	−0.72	0.099
Phubbing → Relationship satisfaction	0.29	0.67	0.124

Regarding the direct effects of the basic psychological needs on phubbing, an increase of one unit in the satisfaction of the need for love/belonging led to a decrease of −0.07 (*β* = −0.10) in phubbing, and an increase of one unit in the satisfaction the need for freedom led to a decrease of −0.15 (*β* = −0.16) in phubbing.

Examining the direct effects of the basic psychological needs on social media addiction, an increase of one unit in the satisfaction of the need for freedom caused an increase of 0.15 (*β* = 0.12) in social media addiction. As for the direct effect of phubbing on social media addiction, an increase of one unit in phubbing scores led to an increase of 1.3 (*β* = 0.97) in social media addiction scores.

Based on the standardized path coefficients, the needs for freedom, fun, and power had a significant small effect on relationship satisfaction, and the need for love/belonging had a significant medium effect on relationship satisfaction. The need for love/belonging and the need for freedom were found to have a small effect on phubbing, and the need for freedom was found to have a small effect on social media addiction. Phubbing was observed to have a large effect on social media addiction (Cohen, 1988).

### Indirect effects

Satisfaction of the basic psychological needs on relationship satisfaction via phubbing and social media addiction was found to have indirect effects on the need for fun (−0.1; *β* = −0.04), the need for freedom (−0.03; *β* = −0.08), the need for love/belonging (0.01; *β* = 0.05), and the need for power (0.00; *β* = 0.03). Those effects were significant but small. The need for fun (0.03; *β* = 0.02), the need for freedom (−0.28; *β* = −0.15), the need for power (0.02; *β* = 0.01), and the need for love/belonging (−0.13; *β* = −0.09) were observed to have indirect effects on social media addiction via phubbing. Indirect effect of phubbing on relationship satisfaction via social media addiction was found to be −0.30 (*β* = −070) (Table 5).

**Table 5:** Bootstrapping results for the indirect effects in the model.

Paths for the model	Coefficient	90% confidence interval	
		Lower limit	Upper limit
Fun → Phubbing → Social media addiction	0.02	−0.07	0.09
Freedom → Phubbing → Social media addiction	−0.15	−0.24	−0.09
Love/belonging → Phubbing → Social media addiction	−0.09	−0.19	−0.01
Power → Phubbing → Social media addiction	0.01	−0.07	0.12
Phubbing → Social media addiction → Relationship satisfaction	−0.70	−4.3	−0.26
Fun → Phubbing → Social media addiction → Relationship satisfaction	−0.04	−0.25	−0.01
Power → Phubbing → Social media addiction → Relationship satisfaction	0.03	−0.00	0.19
Freedom → Phubbing → Social media addiction → Relationship satisfaction	−0.08	−0.28	−0.02
Love/belonging → Phubbing → Social media addiction → Relationship satisfaction	0.05	0.009	0.26

As for the standardized coefficients of the indirect effects, the needs for fun and power had small indirect effects, and the needs for love/belonging and freedom had medium indirect effects on social media addiction via phubbing. Indirect effects of the basic psychological needs on relationship satisfaction via phubbing and social media addiction were found to be small. It was concluded that phubbing had a large indirect effect on relationship satisfaction via social media addiction (Preacher and Kelley, 2011).

Significance of the mediation effects was calculated with the Sobel test. Phubbing was found to be a mediator variable in the correlation between love/belonging of the basic psychological needs and social media addiction (*Z* = −2.84; *p* < 0.05). In addition, phubbing and social media addiction combined had a mediating role in the correlation between love/belonging of the basic psychological needs and relationship satisfaction (*Z* = 2.47; *p* < 0.05).

Phubbing had a mediating role in the correlation between the need for freedom and social media addiction (*Z* = −4.18; *p* < 0.05). Social media addiction and phubbing combined had a mediating role in the correlation between the need for freedom and relationship satisfaction as well (*Z* = 3.12; *p* < 0.05).

Phubbing had a mediating role in the correlation between the need for power and social media addiction (*Z* = −2.63; *p* < 0.05). Moreover, social media addiction and phubbing combined had a mediating role in the correlation between the need for power and relationship satisfaction (*Z* = 2.32; *p* < 0.05).

No mediating roles were found for phubbing in the correlation between the need for fun and social media addiction, and for social media addiction and phubbing combined in the correlation between the need for fun and relationship satisfaction. Social media addiction was found to be a mediator variable in the correlation between phubbing and relationship satisfaction (*Z* = −4.93; *p* < 0.05).

## Discussion

Correlation analyses indicated that the satisfaction of basic psychological needs in romantic relationships was correlated with social media addiction. Expectedly, the needs for love/belonging, power, and freedom were observed to be significantly negatively correlated with social media addiction whereas no significant correlation was found between the need for fun and social media addiction. There is a study which achieved similar results compared to the finding in question (Bozkurt and Bozkurt, 2022). Regarding the direct effects in the model, satisfaction of the need for freedom had a significant effect on social media addiction. When explaining the nature of negative addictions, Glasser (1976) emphasized the importance of meeting the basic needs. Unless the basic psychological needs are met, individuals might resort to addictions such as alcohol, gambling, or substances. In addition, it was suggested that those addictions are addressed under the topic of ineffective mental health behaviors, and individuals could develop psychopathology due to these behaviors (Wubbolding, 2015). In the present study, similarly, social media addiction was reduced with increased satisfaction of the basic psychological needs. However, only the need for freedom had a direct effect on social media addiction in the model established in the study. Other subscales did not have any direct effects, but indirect effects via phubbing. Previous studies have concluded that social media addiction is negatively correlated with some of the subscales competence, autonomy, and relatedness whereas others are not significantly correlated with social media addiction (Yıldırım, 2019; Erdem, 2022).

Significant negative correlations were observed between basic psychological needs and phubbing. According to the other findings of the research, the needs for love/belonging and freedom were significant negative predictors of phubbing. Direct effects of other needs on phubbing were not found to be significant. Phubbing refers to the fact that individuals choose their smartphones over communicating with others in social settings. This can be influenced by social media applications, digital games, and other applications on the smartphone, and FoMO (Chotpitayasunondh and Douglas, 2016; Roberts and David, 2016; Al-Saggaf et al., 2019; Aljasir, 2022). Failure to adequately satisfy basic psychological needs may cause individuals to meet those needs via the applications on their smartphones, which might lead to increased use of smartphones during communication in their close relationships. Similar to the findings of the present research, basic psychological needs were found to be predictors of phubbing (Butt and Arshad, 2021). Satisfaction of basic psychological needs is considered important in avoiding psychopathology and ineffective mental health behaviors (Glasser, 1976; Wubbolding, 2015).

Satisfaction of basic psychological needs in romantic relationships could affect relationship satisfaction. As shown by the correlation values obtained in the research, satisfaction of all basic psychological needs and relationship satisfaction were significantly positively correlated on medium and high levels. The direct effects in the model suggested that satisfaction of basic psychological needs significantly predicted relationship satisfaction. This goes to show the importance of having a person or group to be together emotionally throughout one’s life for meeting those basic needs, as stated by Glasser (1965). Having someone close in one’s life is important to satisfy their needs (Glasser, 1965). In addition, couples could be happy when the need for love/belonging is highly satisfied and satisfaction level of the need for power is low (Glasser and Glasser, 2007). In the present study, the needs for power and freedom significantly predicted relationship satisfaction. This might have been due to the fact that when preparing the items in the Satisfaction of Basic Psychological Needs in Romantic Relationships Scale as part of the research, items were selected to determine how well couples support each other’s needs for power and freedom. There are studies which support the research findings. In previous studies, basic psychological needs predicted relationship satisfaction and the quality of romantic relationship (Kulaber-Demirci, 2019; Sağkal and Özdemir, 2019; Arslan, 2020; Eşici and Özbay, 2020; Güleç, 2020). It might be important for a healthier progression in romantic relationships that couples are aware of each other’s needs and express those needs without lying and blaming each other (Knee et al., 2014).

Examining the indirect effects, the needs for freedom and love/belonging had a significant negative effect on social media addiction via phubbing. The needs for power and fun had a significant positive small effect on social media addiction. The bootstrapping results also indicated that the indirect effects were significant. As shown by the Sobel test performed to determine whether the mediation effect was significant, phubbing was a significant mediator in the correlation between the needs for love/belonging, freedom, and power and social media addiction, but did not have a significant mediating role in the correlation between the need for fun and social media addiction. Overall, it was concluded that phubbing had a mediating role in the correlation between the needs for belonging, freedom, and power of the basic psychological needs and social media addiction.

It was observed that satisfaction levels of basic psychological needs in romantic relationships could lead to negative addictions, and behaviors such as phubbing which might have an adverse impact on communication had a mediating role in that correlation. Social media addiction, video game addiction, FoMO, and Internet addiction are associated with phubbing behavior. Smartphone addiction also has an important effect on phubbing (Suralaga and Pemayun, 2020).

The findings showed that phubbing had a large indirect effect on relationship satisfaction via social media addiction. There are studies in the literature performed on social media addiction, phubbing, and relationship satisfaction. Negative correlations have been observed between phubbing behavior and relationship satisfaction and marriage (Roberts and David, 2016; Chotpitayasunondh and Douglas, 2018a; Chmielik and Blachnio, 2021; David and Roberts, 2021; Wang et al., 2021; Roberts and David, 2022; Thomas et al., 2022; Wang and Zhao, 2022; Yam, 2023). A positive correlation was found between phubbing and relationship satisfaction (Aljasir, 2022). It can trigger phubbing when individuals occupy themselves too much with their smartphones while they are spending time with their romantic partners (David and Roberts, 2021). Social media addiction is one of the addictions that may lead to phubbing behavior. The relevant research finding supports the information above.

When the findings of the research are examined, in the model established, there are differences other than the need for entertainment. Satisfaction of the needs for love/belonging, power and freedom through phubbing and social media. It was found to have an indirect effect on relationship satisfaction. These indirect effects is at a low level. The significance of the indirect effects was determined by the Bootstrapping method. Its significance was calculated with the Sobel test. Model fit values are also excellent and good fit meets the values criteria. Satisfaction of basic psychological needs can play a substantial role on individuals’ happiness. It is important for individuals to be in close relationships to meet those needs. Romantic relationships can be a little more prominent than other close relationships in satisfying the basic psychological needs. In the present study, social media addiction and phubbing were found to be significant mediators in the indirect effect of satisfying the basic psychological needs on relationship satisfaction. This finding supports the ideas stated by Glasser (1965, 1976, 1998) that satisfaction of basic psychological needs would lead individuals to happiness, the romantic partner has a substantial place in such satisfaction, and when failing to satisfy those needs, individuals might develop ineffective mental health behaviors, negative addictions, and psychopathology.

Considering the relationship of addictions with basic psychological needs, it shows the importance of taking basic psychological needs into consideration when addressing addiction in psychological counseling, family counseling and couple therapy practices. While working on the issue of addiction, practices aimed at satisfying the basic psychological needs of individuals can be effective. For this reason, it is important for individuals to have knowledge about choice theory and basic psychological needs, thus gaining awareness about their own needs and choices. Having information about an individual’s choices and basic psychological needs will help them evaluate themselves more healthily in their romantic relationships. In order to increase their satisfaction with the relationship, they will be able to include effective mental health behaviors and positive addictions in their lives instead of addictions.

### Conclusion, limitations and future research

More studies on how satisfaction of basic psychological needs and social media addiction, which is one of the addictions that have emerged with the advancement in technology, affect couple relationships might contribute to more well-based perspectives in the processes of couples and family counseling. In addition, the issue of which needs individuals’ social media addictions help them meet can be addressed. In this way, basic psychological needs-based practices on relationship satisfaction can be addressed by family and couple counselors. There are several studies in the literature that examine the correlation between phubbing and relationship satisfaction. In addition to those studies, addictions including video game addiction, Internet addiction, and social media addiction, which could influence relationship satisfaction as addressed in the present study, can be investigated.

The present study concluded that relationship satisfaction was predicted by the satisfaction of basic psychological needs in romantic relationships. Based on this finding, it could be useful if psychological counselors and other fieldworkers working in couples and family counseling take individuals’ basic psychological needs into consideration when providing pre-marriage counseling services. Glasser (1998) highlighted how it is important for individuals to have knowledge on choice theory before marriage. Individuals could benefit from being aware of their needs both for their relationships and themselves. It was observed in the results of the present study that individuals might choose ineffective mental health behaviors, develop negative addictions, which could negatively affect relationship satisfaction when they fail to satisfy their basic psychological needs adequately. In couples and family therapies or pre-marriage counseling, studying the ineffective mental health behaviors chosen by individuals to satisfy their own psychological needs could contribute to increased relationship satisfaction in marriages and romantic relationships.

Although scales for which validity and reliability studies were performed were utilized in the research, those scales are limited with the structures that they measure. Since the participants were aged between 18 and 30 years, the generalization applied only to that age group, which can be considered among the limitations of the research. For this reason, in future studies, it can be investigated how effective social media, phubbing and basic psychological needs are on the relationship satisfaction of individuals in other age groups.

## Data availability statement

The raw data supporting the conclusions of this article will be made available by the authors, without undue reservation.

## Ethics statement

The studies involving humans were approved by Necmettin Erbakan University Social Sciences and Humanities Ethical Committee. The studies were conducted in accordance with the local legislation and institutional requirements. The participants provided their written informed consent to participate in this study.

## Author contributions

HK: Writing – original draft, Visualization, Resources, Formal analysis. CA: Writing – review & editing, Validation, Supervision, Methodology, Conceptualization.
